# A Novel Peptide Derived from Human Apolipoprotein E Is an Inhibitor of Tumor Growth and Ocular Angiogenesis

**DOI:** 10.1371/journal.pone.0015905

**Published:** 2011-01-06

**Authors:** Partha S. Bhattacharjee, Tashfin S. Huq, Tarun K. Mandal, Richard A. Graves, Syed Muniruzzaman, Christian Clement, Harris E. McFerrin, James M. Hill

**Affiliations:** 1 Department of Biology, Xavier University of Louisiana, New Orleans, Louisiana, United States of America; 2 Department of Ophthalmology, Louisiana State University Health Sciences Center, New Orleans, Louisiana, United States of America; 3 College of Pharmacy, Xavier University of Louisiana, New Orleans, Louisiana, United States of America; 4 Neuroscience Center, Louisiana State University Health Sciences Center, New Orleans, Louisiana, United States of America; 5 Department of Microbiology, Immunology and Parasitology, Louisiana State University Health Sciences Center, New Orleans, Louisiana, United States of America; 6 Department of Pharmacology, Louisiana State University Health Sciences Center, New Orleans, Louisiana, United States of America; University of Florida, United States of America

## Abstract

Angiogenesis is a hallmark of tumor development and metastasis and now a validated target for cancer treatment. We previously reported that a novel dimer peptide (apoEdp) derived from the receptor binding region of human apolipoprotein E (apoE) inhibits virus-induced angiogenesis. However, its role in tumor anti-angiogenesis is unknown. This study demonstrates that apoEdp has anti-angiogenic property *in vivo* through reduction of tumor growth in a mouse model and ocular angiogenesis in a rabbit eye model. Our *in vitro* studies show that apoEdp inhibits human umbilical vein endothelial cell proliferation, migration, invasion and capillary tube formation. We document that apoEdp inhibits vascular endothelial growth factor-induced Flk-1 activation as well as downstream signaling pathways that involve c-Src, Akt, eNOS, FAK, and ERK1/2. These *in vitro* data suggest potential sites of the apoE dipeptide inhibition that could occur *in vivo*.

This is the first evidence that a synthetic dimer peptide mimicking human apoE has anti-angiogenesis functions and could be an anti-tumor drug candidate.

## Introduction

Angiogenesis is defined as the formation of new blood vessels from pre-existing vasculature. Angiogenesis is relevant not only to cancer but also to non-neoplastic diseases including macular degeneration, psoriasis, endometriosis, and arthritis [1], [2]. The growth and metastasis of tumors are critically dependent on angiogenesis [3], [4]. Thus, the inhibition of angiogenesis has become an important therapeutic strategy for cancer [5]. Although the existing anti-angiogenesis therapies have been reported to have less toxicity than conventional chemo or radiotherapy, they are often associated with clinical side effects, and limited tumor regression [6]. Therefore, there has been an increased focus toward development of novel angiogenesis inhibitors and novel approaches to maximize the anti-angiogenic therapies [7].

Human apolipoprotein E (apoE) is one of the most frequently studied proteins known to be involved in lipid metabolism and cardiovascular disorders [8]. Experimental studies on apoE are focused on its receptor binding region, which is located between residues 130–150 and is critical for its biological activity. This receptor binding region of apoE is known to be responsible for binding apoE to the low-density lipoprotein receptor [9]. Within this receptor binding region, residues 142–147, also known as heparin-binding domain, mediate the attachment of apoE to cellular heparan sulfate proteoglycan (HSPG) [10], [11]. HSPG is an integral component of cell surface extracellular matrix that is ubiquitous in nature and plays important roles in the regulation of several aspects of cancer biology, including angiogenesis, tumor progression, and metastasis [12], [13]. Several growth factors including vascular endothelial growth factor (VEGF) and their receptors (VEGFR) bind to HSPG molecules to facilitate cellular and biochemical responses [14]. Thus, molecules having the ability to block these interactions and inhibiting processes crucial to tumor progression are considered as a new class of cancer therapeutics [15], [16]. A tandem-repeat dimer peptide called apoEdp, derived from the apoE residues 141–149, has been reported by others to exhibit anti-infective activity *in vitro* [17], [18] and also in our *in vivo* studies [19], [20]. During our experimental anti-infective studies *in vivo* in mice [19] and rabbit [20] eye models, we recognized that apoEdp inhibited virus-induced corneal angiogenesis. Thus, we have investigated whether apoEdp has the ability to inhibit angiogenesis in two *in vivo* non-infectious models of ocular and tumor angiogenesis. The role of apoEdp as an anti-angiogenic agent is unknown.

VEGF and its endothelial receptors VEGFR has been the subject of great interest in angiogenesis research [21]. Tumor cells secrete VEGF [22], [23]. Endothelial VEGF receptors, particularly VEGF receptor 2(KDR/Flk-1) has been reported to initiate critical signaling pathways through interaction with VEGF, leading to tumor angiogenesis [24]. Targeting the VEGF signaling pathways is a well-known validated approach for anti-tumor therapy [24]. Inhibition of VEGF has been shown to be effective in cancer [25] and ocular angiogenesis [26]. Although VEGF-triggered angiogenesis is not fully understood, groups of signaling molecules such as cSrc [27], [28], FAK [28], Akt [29], ERK1/2 [30] and eNOS [31] have been reported to be involved in VEGF signaling cascades. It has been reported that VEGF treatment of ECs promotes cell survival, proliferation and migration, mainly through the activation of Flk-1 receptor [32], [33]. The activated Flk-1 subsequently binds to c-Src and phosphorylates it [34]. c-Src phosphorylation and c-Src-mediated FAK phosphorylation are essential for the VEGF signaling pathways and in angiogenesis [35]. Reports suggest that c-Src becomes activated following binding to phosphorylated Flk-1 receptor upon VEGF stimulation, with subsequent activation of downstream molecules, including ERK1/2 phosphorylation [36]. VEGF-stimulated EC migration has been reported to result from increased NO production via eNOS phosphorylation [37]. Akt phosphorylation has been reported to be necessary for eNOS activation, endothelial cell (EC) migration and angiogenesis [38]. In this study, we investigated the anti-tumorigenic ability of apoEdp *in vitro* and *in vivo* and examine the underlying cellular and molecular mechanisms. Our *in vitro* results suggest that apoEdp inhibited VEGF-induced human umbilical vein endothelial cell (HUVEC) survival, migration, invasion, and capillary tube formation *in vitro*. The *in vivo* rabbit eye model assay showed that apoEdp suppressed VEGF-induced angiogenesis in a corneal micro-pocket assay. In the mouse xenograft human breast cancer tumor model, apoEdp significantly suppressed the growth of tumor without any observable toxicity to mice. We also examined the signaling pathways related to angiogenesis. The apoEdp selectively blocked the activation of VEGF receptor Flk-1 and the downstream signaling pathways of c-Src, Akt, eNOS, FAK, and ERK1/2 activation. This is the first evidence that a peptide derived from human apoE is a potential anti-angiogenesis and anti-tumor therapeutic agent.

## Results

### ApoEdp affects the viability of endothelial cells but not breast cancer cells (MDA-MB-231)

**Figure 1:**
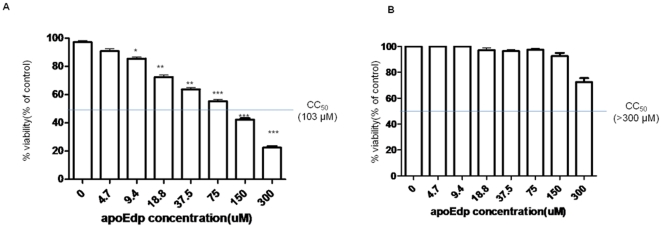
ApoEdp affects HUVEC survival but not MDA-MB-231. **A. HUVEC**: ApoEdp has a significant effect on VEGF-induced HUVEC viability. Cells were treated with apoEdp at the indicated concentrations and incubated with constant 50 ng/mL VEGF for 48 hr. All values were normalized to cell control of no VEGF and no apoEdp. Cell viability values (mean ± SEM) are expressed as % of the value of the VEGF control. **B. MDA-MB-231**: ApoEdp has no effect on MDA-MB-231viability. ApoEdp has no significant effect on MDA-MB-231 survival. Cells were treated with apoEdp at the indicated concentrations and incubated at 37°C for 2 days. Cell viability values (mean ± SEM) are expressed as % of the value of the control (untreated cells). The asterisks indicate significant differences compared with VEGF stimulation. ** *p*<0. 01; and *** *p*<0.001.

To confirm that apoEdp affects cell survival, cell viability was determined using the cell titer 96®AQuous One solution cell proliferation kit. ApoEdp treatment of HUVECs induced a dose-dependent inhibition of cell viability (Fig. 1A). The 50% cytotoxicity (CC_50_) determined in this assay was about 103 µM. To determine whether apoEdp suppresses tumor growth by directly affecting tumor cells, we performed a cell viability assay using human breast cancer MDA-MB-231 cell line *in vitro* and cell titer 96®AQuous One solution cell proliferation kit. As shown in Fig. 1B, the 50% cytotoxic dose of apoEdp against breast cancer cells was >300 µM, suggesting that apoEdp has no significant direct effect on tumor cell proliferation.

### ApoEdp inhibits endothelial cell migration/wound healing

**Figure 2:**
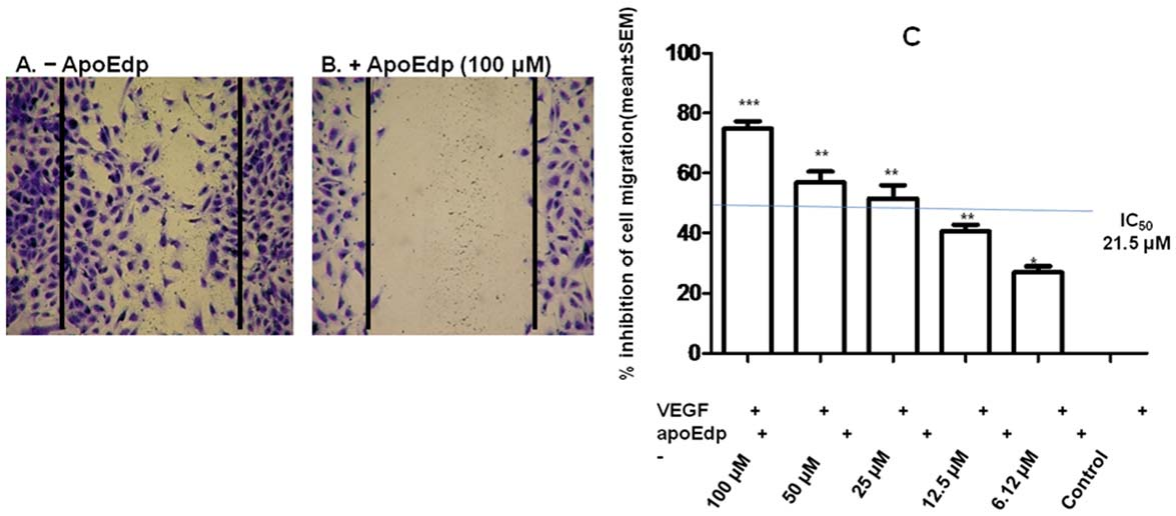
ApoEdp inhibits wound-healing migration *in vitro*. Monolayers of HUVECs were scraped and incubated in medium containing 50 ng/mL VEGF in the presence or absence of various concentrations of apoEdp. VEGF stimulated the migration of HUVECs in the scraped area. Representative photomicrographs of cells treated with (A) VEGF alone or with (B) VEGF and apoEdp together are shown. Solid lines indicate the initial scraping. (C) ApoEdp significantly inhibited the migration of HUVEC to the wounded area. The bar diagram is the quantitative measurement of cell migration inside the scraped area. The data shown are representative of three independent experiments. ** *p*<0. 01; and *** *p*<0.001.

An important angiogenic phenotype of ECs induced by VEGF is cell migration. The major aspects of VEGF action on ECs are its ability to act as a chemoattractant and to stimulate the angiogenic morphogenesis. We therefore examined the ability of apoEdp to inhibit VEGF-induced HUVEC migration, since such migration is relevant to angiogenesis and hence to tumor growth and metastasis. Cell migration was examined after simulation of an artificial wound by perturbation of a cell monolayer using a 1-mL pipette tip. While using the wound healing assay to measure changes in cell migration (Fig. 2A–B), we found that the inhibitory effect of apoEdp on HUVEC migration is dose dependent at the range of 6–100 µM; the 50% inhibitory concentration (IC_50_) for this assay is approximately 21.5 µM. (Fig. 2C).

### ApoEdp inhibits capillary tube formation in vitro

**Figure 3:**
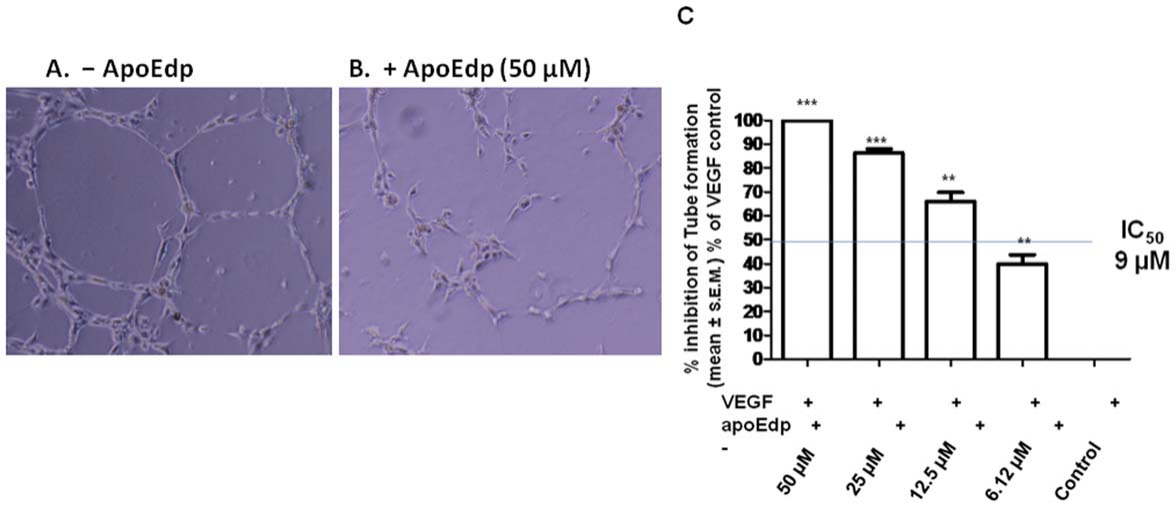
ApoEdp inhibited capillary tubule formation (*in vitro* angiogenesis). About 4×10^4^ (per well) HUVECs in medium containing 50 ng/mL of VEGF were plated in 24-well plates previously coated with growth factor-reduced matrigel, and incubated for 12–16 hr at 37°C in the absence or presence of apoEdp. Tubular structures were quantitated by manual counting under low power fields. Representative photomicrographs of tubule formation in the (A) VEGF control and (B) apoEdp-treated wells are shown. (C) “Percentage (%) of inhibition” is the mean number (± SEM) of tubules expressed as a proportion of that in the VEGF control group. ** p<0.01 and *** p<0.001.

The cellular mechanisms of angiogenesis involve motility and alignment of ECs to form a capillary tube-like network on matrigel [31]. Thus, we assessed whether or not apoEdp impaired EC differentiation in the tube formation assay as described in the Materials and Methods. The numbers of tubular structures (Fig. 3A) were quantitatively analyzed. As shown in Fig. 3B, apoEdp significantly inhibited the ability of HUVEC capillary tube formation compared to VEGF control (VEGF only, no peptide) in a concentration-dependent manner from the range of 6–50 µM; the IC_50_ for this assay was 9 µM. “Percentage (%) of inhibition” in Fig. 3C is the mean number (± SEM) of tubules expressed as a proportion of that in the VEGF control group. ** p<0.01 and *** p<0.001.

### ApoEdp inhibits endothelial cell invasion

**Figure 4:**
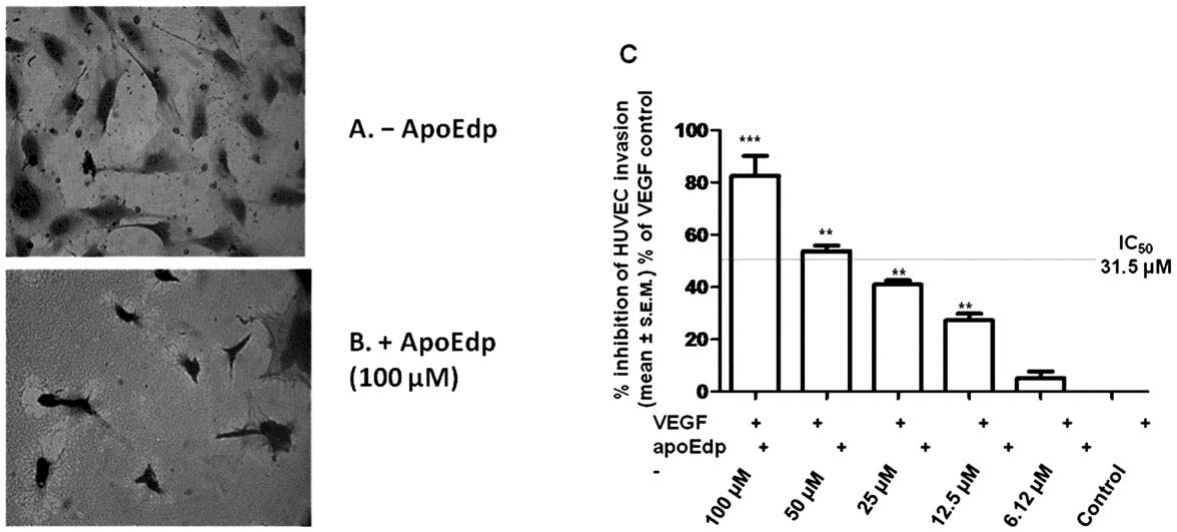
ApoEdp inhibits endothelial cell invasion. About 4×10^4^ HUVECs were placed into each insert of Boyden chamber (0.8-µm pore size) (BD Biosciences) of 24-well transwell plate. The bottom chambers were filled with 600 µL medium supplemented with 50 ng/mL VEGF. The top chamber was seeded with approximately 4×10^4^ HUVEC cells/well in 100 µL containing different concentrations of apoEdp and incubated at 37°C for 16 hs. HUVEC cells were then fixed and stained with H&E and counted under the microscope. Representative photographs of (A) VEGF-treated and (B) apoEdp-treated membranes are shown. The results show that the (C) HUVECs migrating across the transwell membrane are significantly suppressed by the peptide apoEdp in a dose-dependent manner. **p<0.01 and ***p<0.001.

The complex process of new blood vessel formation involves migration and invasion of ECs. The inhibition of EC motility through migration and invasion could thus be expected to affect the angiogenic process. To evaluate the ability of apoEdp in blocking VEGF-induced HUVEC migration and invasion, we used a Boyden chamber transwell assay (Materials and Methods). We quantified the invasion of HUVECs across the transwell. Cells which invaded across the membrane were stained with hematoxylin and eosin stain and counted (Fig. 4A–B). Compared to VEGF-induced control, apoEdp peptide significantly inhibited VEGF-induced cell migration and invasion at a dose range of 6–100 µM; the IC_50_ for this assay was about 31.5 µM (Fig. 4C). All values were normalized to cell numbers of transwells exposed to EBM containing 1% FBS without apoEdp and VEGF.

### ApoEdp inhibits in vivo angiogenesis in rabbit corneal micropocket assay

**Figure 5:**
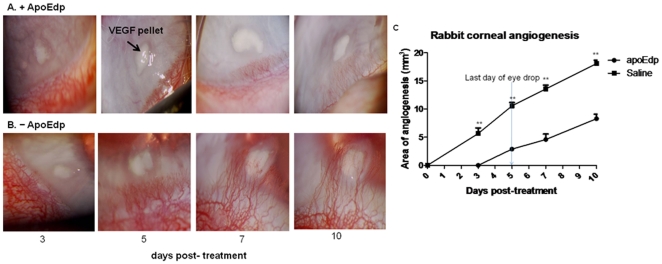
ApoEdp inhibits angiogenesis in rabbit corneal micropocket assay. Slow-releasing micro pellets (500 µm×500 µm) containing 160 ng of VEGF were corneally implanted in New Zealand white rabbits (1.5–2.5 kg). The pellets were positioned about 2.0 mm from the corneal limbus. Treatment began one day post-implantation (PI) and continued for five consecutive days. The right eye of all 5 rabbits was treated using 1% peptide apoEdp, and left eye of the same rabbit received saline drops as mock treatment. Each eye drop (50 µL) was applied topically 5 times per day every 2 hr starting at 8 AM and ending at 4 PM. Data and photos were obtained on days 3 through 10 after pellet implantation. (A)The area of neovascular response, vessel length, and clock hours of new blood vessel of rabbits in each group were calculated according to the formula Area (mm^2^)  = C/12×3.1416 [r^2^−(r−L)^ 2^] where C  =  the number of clock hours at the limbus involved in the neovascular response, L  =  length of the longest neovascular pedicle from the limbus onto anterior cornea, and r  =  radius of the cornea. (B). ApoEdp significan.tly inhibited VEGF-induced angiogenesis in rabbit corneal micro pocket assay. **p<0.01 and ***p<0.001.

To study the *in vivo* anti-angiogenic effect of apoEdp, we assessed its inhibitory effect on VEGF-induced corneal micro-pocket assay in rabbit eye model. The slow release micropellet (500 µm×500 µm) of 0.4 g of Compritol 888 ATO (Gattefosse) combined with 0.1 g of Squalane oil (Sigma) and 20 mg/mL L-α-phosphatidylcholine containing saline or 160 ng of VEGF were surgically implanted in the corneal micropocket created in the avascular area of rabbit cornea (Fig. 5A–B). Topical application of eye drops containing 1% apoEdp significantly inhibited VEGF-induced angiogenesis compared to saline treated eyes (Fig. 5C). Corneal micro-pocket application of mock-pellets containing saline did not induce angiogenesis throughout the examination period.

### ApoEdp suppresses tumor growth in Nude (nu/nu) mice xenograft model

**Figure 6:**
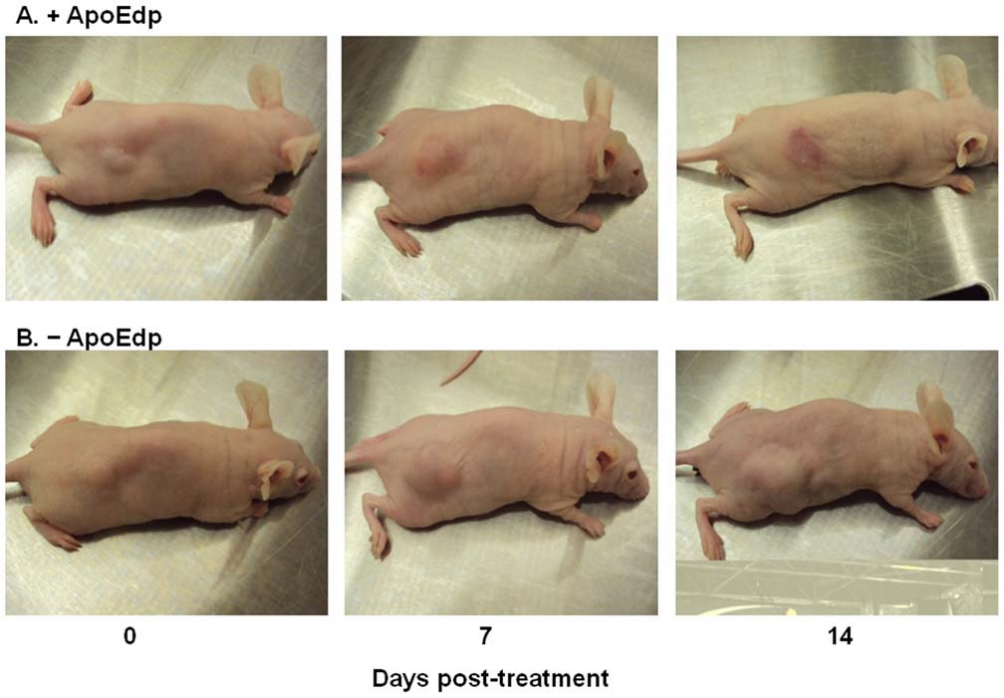
ApoEdp inhibits tumor growth *in vivo.* MDA-MB-231 breast cancer cells were used for anti-tumor studies. A suspension of 3×10^6^ cells in 0.1 mL of PBS was injected in the right dorsal flank. Tumors were monitored weekly in two diameters with digital calipers. Tumor volumes were determined using A×B^2^×0.52 (where A is the longest and B is the shortest diameter). Tumors were allowed to grow to ∼100 mm^3^ and mice were randomized. Single intralesional administration daily of PBS or 40 mg/kg/day of apoEdp for 3 consecutive days was performed and then stopped. Representative photographs of mouse xenograft treated with (A) apoEdp or PBS are shown. (B) ApoEdp treatment significantly inhibited tumor growth throughout the examination period. *p<0.05, and ***p<0.001.

In order to assess the *in vivo* effect of apoEdp on tumor growth, we established subcutaneous human breast cancer MDA-MB-231 tumor xenografts in nude mice. Once tumor size became ∼100 mm^3^ in approximately 10 days, mice were randomly divided into two groups, and treated with PBS or apoEdp at 40 mg/kg/day for three consecutive days. Representative photographs of apoEdp- or PBS-treated mouse xenografts are shown in Fig. 6A–B. ApoEdp treatment significantly inhibited tumor growth compared to PBS- treated controls (Fig. 6C). There was no sign of observable toxicity and no significant body weight differences between the two groups of mice (data not shown) were detected during drug treatment.

### ApoEdp inhibits VEGF-induced signaling pathways

**Figure 7:**
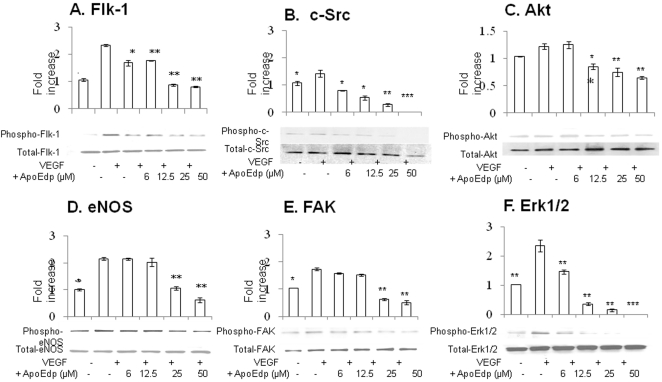
Inhibition by apoEdp of VEGF-induced Flk-1. HUVECs were pre-treated with apoEdp at the indicated concentrations for 1 hr; VEGF (10 ng/mL) added and incubated at 37°C for 10 min, to measure the phosphorylation of Flk-1(7-A), c-Src(7-B), Akt(7-C), eNOS(7-D), FAK(7-E), and Erk1/2(7-F). Equal loading of total proteins was also measured as described in Materials and Methods. Values were normalized by arbitrarily setting the densitometry of control cell signals 1.0 (mean ± SEM, n = 5). The asterisks indicate significant differences compared with VEGF stimulation (*p<0.05, **p<0.01 and ***p<0.001).

Our results suggest that apoEdp significantly inhibited VEGF-induced phosphorylation of Flk1 (Fig. 7-A), c-Src (Fig. 7-B), Akt (Fig. 7-C), eNOS (Fig. 7-D), FAK (Fig. 7-E), and Erk1/2 (Fig. 7-F) in a concentration-dependent manner.

## Discussion

Cellular processes of tumor angiogenesis and metastasis involve multiple steps including EC proliferation, migration, and differentiation through tube formation, invasion and neo-angiogenesis [1]–[4]. Peptides or compounds that inhibit any of the multistep processes will lead to the disruption of angiogenesis and can serve as potential candidates for therapeutic intervention against angiogenesis [2].

Human apoE is a protein constituent of different plasma lipoproteins and serves as a high-affinity ligand for several receptors. By interacting with its receptors, apoE mediates the clearance of different lipoproteins from the circulation [39]. Absence or structural mutations of apoE cause significant disorders in lipid metabolism, cardiovascular disease, and Alzheimer's disease [8]. Specific peptide sequences derived from the receptor-binding region of human apoE have been previously reported by us [19], [20] and others [17], [18], [40], [41] to have anti-infective and anti-inflammatory properties. No reports are available about the effect of a peptide derived from human apoE against tumor angiogenesis.

Apolipoprotein E shares a series of features with another polymorphic apolipoprotein, apolipoprotein A [apoA] [42], [43]. ApoA consists of tandemly repeated kringle domains that closely resemble plasminogen kringle 4 (KIV) [44]. The apoA kringle domains have been reported to inhibit angiogenesis *in vitro* [45] and suppress tumor growth *in vivo* [46], [47]. ApoA binds to the same receptors as apoE [48], [49]. Because of the similarities between apoE and apoA in receptor binding, we hypothesized that the apoE receptor domain is also associated with angiogenesis and tumor growth.

ApoEdp sequence is derived from a region (ApoE_142–149_) of apoE known to bind cell surface heparan sulfate proteoglycan (HSPG) of extracellular matrix (ECM) to exert its biological functions [50], [51]. HSPG-based antiangiogenic therapies have been previously reported to be a method of choice in cases where tumor angiogenesis depends on cell-surface HSPG [52], [53]. The oligosaccharide component of HSPG (heparan sulfate) and its mimetics have been reported to function by blocking these interactions and inhibiting processes crucial to tumor progression and, therefore, present a promising approach for new cancer therapeutics [52], [53]. One recent report [14] suggests that a HSPG-binding peptide derived from VEGF inhibits tumor growth by blocking angiogenesis in murine model. No reports are available on whether a HSPG-binding peptide derived from the receptor-binding region of human apoE E can inhibit tumor growth by blocking angiogenesis.

Because tumor growth is dependent on tumor angiogenesis, and angiogenesis inhibition is a novel therapeutic modality toward controlling solid tumors, we demonstrate that apoEdp peptide significantly inhibits tumor growth in nude mice with MDA-MB-231 tumor cells. However, unlike HUVEC, the peptide has no significant effect on the growth of MDA-MB-231 cells *in vitro* even at a high concentration (300 µM). At the same time, apoEdp peptide inhibited HUVEC cell migration and tube formation at low concentrations in a wound-healing/migration assay, Boyden chamber migration assay, and tube formation assays. The *in vivo* effects of apoEdp on suppression of tumor growth suggest that apoEdp is anti-tumorigenic by directly arresting EC growth and indirectly starving tumor cell proliferation.

One possible mechanism of the anti-angiogenic property of apoEdp could be related to our previous findings of the ability of apoEdp is to interfere with herpes virus-induced angiogenesis via inhibition of VEGF [19]. VEGF receptor-2 (Flk-1) is the primary receptor in the VEGF signaling pathway that regulates angiogenesis [54]. In the present study, we report that apoEdp inhibits VEGF-mediated receptor (VEGFR2/Flk-1) activation and downstream signaling (c-Src, FAK, Akt, eNOS, Erk1/2) pathways of angiogenesis pertinent to tumor development and progression.

**Figure 8:**
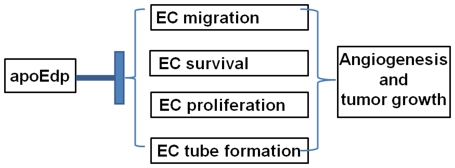
A working model to explain anti-angiogenic activity of apoEdp driven by VEGF. ApoEdp peptide suppresses tumor growth through inhibition of VEGF-induced EC proliferation, migration, invasion, and capillary tube formation.

In conclusion, we showed for the first time that an 18-amino acid dimer peptide (apoEdp) from human apolipoprotein E has potential multi-targeted anti-angiogenic properties (Fig. 8). We highlighted the roles of the apoEdp peptide in the inhibition of tumor growth. *In vitro*, apoEdp inhibits EC survival, proliferation, migration, and capillary tube formation. Angiogenesis inhibition by apoEdp is regulated by selectively blocking VEGF-induced Flk-1 receptor activation and downstream signaling pathway of c-Src-Akt-eNOS, FAK, and Erk1/2. Our findings in the present study could shed more light on the pharmacological activity of apoEdp and its potential for clinical use as an anti-angiogenic agent for the suppression of tumor growth and metastasis.

## Materials and Methods

### Ethics Statement

The Xavier University of Louisiana Institutional Animal Care and Use Committee approved this study (protocol 02-182009-1B), as did the Louisiana State University Health Sciences Center in New Orleans IACUC (protocol 2652). All studies were performed in accordance with the Institute of Laboratory Animal Research (NIH, Bethesda, MD) Guide for the Care and Use of Laboratory Animals.

### Cells and peptides

HUVEC cells were obtained from Lonza (Walkersville, MD) and were maintained in EBM-2 (Lonza) supplemented with 10% fetal bovine serum (FBS) and growth factors (bullet kit, Lonza). HUVEC cells were used between passages 2–6. Human breast cancer cell MDA-MB-231 was obtained from the American Type Tissue Collection and cultured in Dulbecco's Modified Eagle's medium supplemented with 10% fetal bovine serum, 0.1 mg/mL streptomycin, and 25 U/mL penicillin. The apoE mimetic peptide (apoEdp) was synthesized (Genemed, Arlington, TX) with a purity of greater than 95%. The 18 amino acid (Ac-LRKLRKRLLLRKLRKRLL-amide) tandem-repeat dimer peptide (apoEdp) was derived from the human apolipoprotein E receptor-binding region between residues 141 and 149, as described previously [1], [2], [4].

### Cell survival assays

#### VEGF-stimulated endothelial cell survival assay

The effect of apoEdp on ED survival was evaluated. Passages 2 to 5 HUVECs were grown to sub-confluency in EBM2 medium (Lonza) containing 10% FBS, EC growth supplement (bullet kit, Lonza). The cells were seeded in 96-well plates at 10,000 per well in EBM2 medium containing growth supplements (bullet kit; Lonza) and 10% FBS. The next day, cells were starved for 18 hrs in EBM2 + 1% FBS without any growth supplement and then incubated with apoEdp in various concentrations. One hour later, 50 ng/mL of VEGF was introduced into the assay. Two days later, cell numbers were determined using the CellTiter96 AQueous One solution cell proliferation kit (Promega, Madison, WI).

#### Breast cancer cell survival assay

Cell survival studies were carried out using the CellTiter96 AQueous One solution cell survival assay. Briefly, cells were plated at about 5,000 MDA-MB-231 cells/well in a 96-well plate and allowed to adhere to the plate with different concentrations of peptides (6–300 µM). The cells were incubated for 48 hr and then the AQueous One solution was added to the samples and measured at 490 nm.

#### Wound-healing/migration assay

To determine the effect of apoEdp on EC migration induced by VEGF, wound-healing assays were performed using HUVEC cells. Cells were allowed to grow to confluency on 6-well plates and washed twice with PBS. Monolayer cells were wounded by scratching with 1-mL pipette tip and washed 3 times with PBS and incubated for 8 h in EBM-2 without any growth supplements except 1% FBS. Following 8 hr incubation, cells were supplemented with 1% FBS and 50 ng/mL of VEGF, either in the presence or absence of the apoEdp (6–100 µM). Control HUVEC cultures were incubated in EBM-2 plus 1% FBS in the absence of VEGF. Migration of the cells into the wounded area was photographed with an Olympus U-RLF-T microscope and counted. All values were normalized to control without VEGF and apoEdp containing wells.

#### HUVEC matrigel tubule formation assay

Matrigel (Sigma Aldrich, St Louis, MO) was thawed overnight on ice. Each well of 24-well plates was coated at 4°C with 300 µL matrigel and incubated at 37°C for 30 min. HUVECs were harvested, and approximately 4×10^4^ cells/well were seeded in 1 mL medium with various concentrations of apoEdp peptide and with/without 50 ng/mL of VEGF. After 12–16 hr, microtubule formation was assessed with an inverted photomicroscope and the images were photographed using Olympus U-RLF-T microscope. Manual counting at low power fields was used to quantitate tubular structures and percentage of inhibition was expressed using VEGF control wells as 100%.

#### HUVEC cell migration/invasion assay

Boyden chamber migration/invasion assays were performed. 24-well transwell (BD Biosciences, San Jose, CA) migration chambers having an 8-µm pore size were used. The transwells were placed in the 48-well plate. The bottom chambers were filled with 600 µL of medium supplemented with/without 50 ng/mL VEGF. The top chamber was seeded with approximately 4×10^4^ HUVEC cells/well in 100 µL containing different concentrations of apoEdp peptide. Cells were allowed to migrate for 16 hr at 37°C. Following incubation, cotton swabs were used to gently scrape the cells on the top surface of the membrane. After scraping of top surface, cells on the bottom side of the membrane were fixed with 10% buffered formalin for 1 hr, washed 3–5 times with PBS, and then stained with hematoxylin and eosin. The cells were then de-stained in PBS, and the transwell membrane was left to air dry at room temperature. Transwell invaded cells were counted using an inverted microscope. Three independent areas per filter were counted, and the mean ± SEM number of migrated cells was calculated.

#### Rabbit corneal micropocket assay

Corneal micropockets were created with a modified von Graefe cataract knife in eyes of each 2–3 lbs NZ white rabbit. A micropellet (500 µm×500 µm) of 0.4 g of Compritol 888 ATO (Gattefosse) combined with 0.1 g of Squalane oil (Sigma) and 20 mg/mL L-α-phosphatidylcholine containing saline or 160 ng of VEGF was implanted into each corneal pocket, positioned about 2 mm from the corneal limbus. The right eye was implanted with a VEGF-containing pellet and the left eye of the same rabbit was implanted with a saline-containing pellet. A group of 5 rabbits was treated with 1% apoEdp and another group of 5 rabbits was treated with saline as eye drops. Treatment started one day post-implantation (PI) and continued for five consecutive days. Each eye drop (50 µL) was applied topically 5 times per day every 2 hr starting at 8 AM and ending at 4 PM. Data and photos obtained on days 3 thru 10 after pellet implantation are provided. The area of neovascular response, vessel length, and clock hours of new blood vessels of the rabbits in each group were calculated according to the formula [55].

Area (mm^2^)  = C/12×3.1416 [r^2^−(r−L)^2^] where, C  =  the number of clock hours at the limbus involved in the neovascular response, L  =  length of the longest neovascular pedicle from the limbus onto anterior cornea, and r  =  radius of the cornea.

#### Xenograft tumor growth assay in nude mice

Female *nu/nu* mice (8–12 weeks old, weighing ∼20 g) were obtained from Charles River Laboratories (Harlan, Indianapolis, IN). Mice were weighed, coded, and divided into experimental groups at random (n = 5 to 6). About 3×10^6^ MDA-MB-231 cells in 100 µL medium were injected subcutaneously into the right sides of the dorsal area. Peptides were intralesionally injected at the dose of 40 mg/kg/day. The dose level was selected based upon pilot experiment of safety and efficacy of apoEdp in xenograft mice tumor model (data not shown). Control groups were injected only with sterile PBS. The growth of the tumor xenograft was evaluated by determining the tumor volume using digital calipers every 7 days. Tumor volume was calculated [14] using the relationship A×B^2^×0.52, where A is the longest diameter and B the shortest. Mice were euthanized 2 weeks after treatment.

### Western blot analysis of angiogenic signaling molecules

Confluent HUVECs were grown in EBM-2 supplemented with 1% FBS for 24 hr and the medium was replaced with fresh low serum (1% FBS) media in the presence or absence of varying concentrations of apoEdp (6–50 µM). After 1 hr, VEGF was added to a concentration of 10 ng/mL and incubated for 10 min so that the phosphorylated forms of angiogenic signaling molecules could be detected. The cells were then lysed, quantified for protein concentration, and separated on 4–20% pre-cast SDS–PAGE gels. Western blots of control and treated lysates were performed with antibodies against the phospho form of Flk-1, c-Src, FAK, ERK1/2, Akt, and eNOS. To show that equal amounts of protein have been loaded, antibodies against total Flk-1, c-Src, FAK, ERK1/2, Akt, and eNOS were used. All primary and secondary antibodies were obtained from Santa Cruz Biotechnology (Santa Cruz, CA).

### Statistical analysis

Result values were expressed as means and SEM, and the significance was established by Student's *t* test. In all analyses, the level of statistical significance was more than 95% confidence level (p, 0.05). *, **, or *** means p<0.05, p<0.01 or p<0.001, respectively.
